# Cardiac MRI and Echocardiography for Early Diagnosis of Cardiomyopathy Among Boys With Duchenne Muscular Dystrophy: A Cross-Sectional Study

**DOI:** 10.3389/fped.2022.818608

**Published:** 2022-03-14

**Authors:** Nidhi Prakash, Renu Suthar, Bhupendra Kumar Sihag, Uma Debi, Rohit Manoj Kumar, Naveen Sankhyan

**Affiliations:** ^1^Pediatric Neurology Unit, Department of Pediatrics, Post Graduate Institute of Medical Education and Research (PGIMER), Chandigarh, India; ^2^Department of Cardiology, Post Graduate Institute of Medical Education and Research (PGIMER), Chandigarh, India; ^3^Department of Radiodiagnosis, Post Graduate Institute of Medical Education and Research (PGIMER), Chandigarh, India

**Keywords:** Duchenne muscular dystrophy (DMD), cardiomyopathy, echocardiography, cardiac MRI (CMRI), ejection fraction

## Abstract

**Background:**

Cardiomyopathy is an important cause of morbidity and mortality in boys with Duchenne muscular dystrophy (DMD). Early diagnosis is a prerequisite for timely institution of cardioprotective therapies.

**Objective:**

We compared cardiac MRI (CMRI) with transthoracic echocardiography (TTE) including tissue Doppler imaging (TDI) and speckle tracking echocardiography (STE) for diagnosis of cardiomyopathy in early ambulatory boys with DMD.

**Methodology:**

This cross-sectional study was conducted between June 2018 and December 2020. Consecutive boys between 7 and 15 years of age with DMD were enrolled. Percentage ejection fraction (EF), fractional shortening, wall motion abnormalities, early diastolic mitral annulus velocity (Ea), medial mitral annulus ratio (E/Ea), and global strain were measured with STE. CMRI-derived EF, segmental hypokinesia, and late gadolinium enhancement (LGE) were studied and compared.

**Results:**

A total of 38 ambulatory boys with DMD were enrolled. The mean age was 8.8 ± 1.6 years, and none had clinical features suggestive of cardiac dysfunction. In the TTE, EF was ≤55% in 5 (15%), FS was ≤28% in 3 (9%), and one each had left ventricular wall thinning and wall hypokinesia. In TDI, none had diastolic dysfunction, and STE showed reduced global strain of < 18% in 3 (9%) boys. CMRI-derived EF was ≤55% in 20 (53%) boys and CMRI showed the presence of left ventricular wall hypokinesia in 9 (24%) and LGE in 4 (11%) boys.

**Conclusion:**

Cardiomyopathy remains clinically asymptomatic among early ambulatory boys with DMD. A significantly higher percentage of boys revealed early features of DMD-related cardiomyopathy in CMRI in comparison with echocardiography.

## Introduction

Duchenne muscular dystrophy (DMD) is an X-linked recessive disorder characterized by progressive and irreversible loss of muscles function. The incidence of DMD is 1 in 5,000 male newborns, and prevalence is 6 cases in 100,000 males (1). DMD is secondary to lack of dystrophin protein; a large membrane-associated protein expresses in striated muscles, brain, and heart (2, 3). Natural history studies of DMD revealed that boys with DMD develop progressive weakness and become non-ambulatory by the second decade of life (4). Later, these children develop progressive scoliosis and respiratory insufficiency. Cardiac involvement manifests with dilated cardiomyopathy, congestive cardiac failure, and arrhythmias.

The incidence of DMD-related cardiomyopathy increases with age, affecting one third of patients by 14 years and is universal by 18 years (2). DMD-related cardiomyopathy has a significantly prolonged subclinical phase of myocardial fibrosis that starts early in the course of the disease, but eventually leads to overt cardiac failure by the second decade (1). Because of marked physical limitation, DMD-related cardiomyopathy remains clinically silent (2, 5).

Transthoracic echocardiography (TTE) is the most frequently used diagnostic modality for DMD-related cardiomyopathy, though systolic dysfunction is rarely detectable before 10 years of age (6). Recent studies have shown improvement in the early diagnosis of DMD-related cardiomyopathy with newer TTE techniques such as Tissue Doppler imaging (TDI), and 2D-speckle tracking echocardiography (STE) (7). Recently, cardiac MRI (CMRI) has been recommended for diagnosis of DMD-related cardiomyopathy (2, 6, 8–10). In this study, we evaluated the comparative efficacy of CMRI and TTE, including TDI and STE, for early diagnosis of cardiomyopathy in young boys with DMD.

## Methodology

This cross-sectional study was conducted in the Pediatric Neurology Clinic of a tertiary care research institute between July 2018 and December 2020. Consecutive ambulatory boys between 7 and 15 years of age presenting with a genetic or muscle biopsy confirmed diagnosis of DMD were included in the study. Boys with coexisting autism, intellectual disability, hypersensitivity to contrast agent, and claustrophobia to MRI were excluded. Written informed consent was taken from the parents prior to enrollment, and assent from the child was taken. The study was approved by the IEC No.12087/PG-2Trg/2018/6093-94 dated 30-1-2019.

Baseline demographic, clinical information, and investigations were recorded in a predesigned case record form (CRF). Functional status at enrollment was assessed by the 6-min walk test (6 MWT) and timed function tests (TFTs) including 10-meter walk time, Gower's time, four stairs ascent and descent time, and upper limb Brook's and lower limb Vigno's scores. Resting heart rate (tachycardia > 110 per min resting heart rate), blood pressure, postural hypotension, and need for respiratory support (bilevel positive airway pressure/continuous positive airway pressure) were recorded.

All patients had standard 12-lead electrocardiograms (filter range, 0.05–100 Hz) with a paper speed of 25 mm/s. Right ventricular pathology was considered in presence of R waves > 4 mm in lead V1, and R/S ratio > 1 in lead V1 and V2. Left ventricular pathology was considered in the presence of deep and narrow Q waves in lead V5 and V6 (11, 12).

Trans thoracic echocardiography (TTE) was done by two expert pediatric cardiologists (RMK, BKS) with an EPIQ7C Philips Machine. TDI and STE were also performed in the same setting, and details were recorded in a prestructured proforma. TTE parameters included ejection fraction (EF), percentage fractional shortening (FS), left ventricular internal diameter in diastole (LVIDd), mitral valve E-wave velocity, presence of LV akinesia/hypokinesia, and thinning behind posterior mitral leaflet. The left ventricular EF was obtained with the biplane Simpson's method, and FS was calculated on M-mode modality. According to echocardiography, left ventricular systolic dysfunction was considered in the presence of EF < 55% and or FS of <28%. Left ventricular internal diameter of (LVID) > 2SD corrected for age and body surface area is defined as dilated cardiomyopathy. Diastolic function was studied by mitral early inflow Doppler velocity (E wave), early-to-late (A wave) inflow Doppler velocity ratio (E/A), medial and lateral mitral annular tissue Doppler imaging (TDI), early inflow velocity (E′), and E/E′ ratio. Early subclinical cardiac changes were studied with STE in apical four-chamber view (for longitudinal measurements) and the parasternal short-axis view at the level of the midpapillary muscles (for circumferential measurements). End diastolic volume (ml/m^2^), end systolic volume (ml/m^2^), and average peak systolic circumferential strain were calculated. Global longitudinal strain (GLS) was measured by STE. GLS < 16% was considered abnormal, 16–18% was borderline strain and >18% strain was considered normal (13–16).

CMRI appointments were scheduled within 2–4 weeks of clinical and echocardiographic evaluation. CMRI was performed on a 3T MRI scanner (Philips Ingenia) with a dedicated phased array surface coil. The MRI protocol included 3-D balanced turbo-field-echo (BTFE) sequences including LGE. LGE images were acquired ~15 min after dynamic perfusion sequence after calculating the TI of myocardium using phase inversion recovery sequence. All images were acquired by parallel imaging technique—sensitivity encoding (SENSE). Segment-wise analysis of the left ventricle was done for perfusion defect in first-pass gadolinium dynamic sequence, end diastolic thickness, and LGE to look for contrast retention. Regions were calculated as per 17-segment AHA model leaving the apical segment as it is supplied by all the three major epicardial coronary arteries (17). Segments of the left ventricle alone were considered for analysis.

Regional wall motion anomalies were calculated using the Likert scale, with score 1 defined as normokinesia or hyperkinesia, score 2 as mild hypokinesia, score 3 as moderate hypokinesia, score 4 as severe hypokinesia, and score 5 as akinesia/ dyskinesia (paradoxical movement of myocardial segment in systole). For LGE analysis, if any LV segment showed myocardial enhancement, it was considered as LGE positivity (LGE+). The number of segments showing LGE+ were calculated according to the American Heart Association 16-segment model (18).

The extent of transmural infarction within each of the segment (expressed as percentage of the total segmental area) was graded using the following scale: Grade 0: no enhancement, Grade I: 25% tissue involvement, Grade II: 26% and more. The CMRI required ~45 min−1 h time and were performed without sedation. Gadolinium MRI contrast was used at a dose of 0.2 ml/kg. CMRI was analyzed by the radiologist (UD) for the presence of cardiomyopathy, areas involved, and presence of LGE and ejection fraction.

DMD-related cardiomyopathy was classified according to the classification proposed by Fayssoil et al. in 2017 based on clinical, echocardiographic, and CMRI findings (1). Stage 1 is asymptomatic with EF > 55% and negative CMRI LGE, stage 2 is tachycardia and EF between 45 to 55%, stage 3 is peripheral limb edema and EF between 35 and 45%, and stage 4 is clinically symptomatic with anasarca and EF < 35% and CMRI shows diffuse LGE presence (1). As a unit protocol, boys with DMD > 6 years of age receive steroids (deflazacort at 0.9 mg/kg/day) and angiotensin-converting enzyme (ACE) inhibitors (enalapril/perindopril) at ≥10 years of age (19).

### Statistical Analysis

#### Sample Size Calculation

Prevalence of cardiomyopathy among boys with DMD at 10 years of age is about 10% according to echocardiography-based studies, and about 50% boys may show evidence of cardiomyopathy in CMRI-based studies (20–22). With a probability of 40% higher detection of cardiomyopathy with CMRI in comparison with conventional 2D-echocardiography, and an alpha error of 0.05 and 80% power, the sample size was estimated to be 36 patients.

Data were recorded on a CRF and entered in Microsoft excel sheet and analyzed with Statistical Package for Social Sciences (SPSS) for Windows version 23 (SPSS Inc., Chicago, IL, USA). Normal distribution of the continuous variables was established with Kolmogorov–Smirnov test and Shapiro–Wilk test. The continuous variables were presented as mean ± standard deviation or median (IQR) based on the distribution, while qualitative data were presented as frequency and percentage (%) and compared using independent T-test/Man–Whitney U test, and chi-square test, or Fisher's exact tests. For the correlation between the CMRI and ECHO-derived parameters, functional assessment was done with Spearman or Pearson's correlation test. A *p*-value of <0.05 was considered statistically significant.

## Results

**Figure 1:**
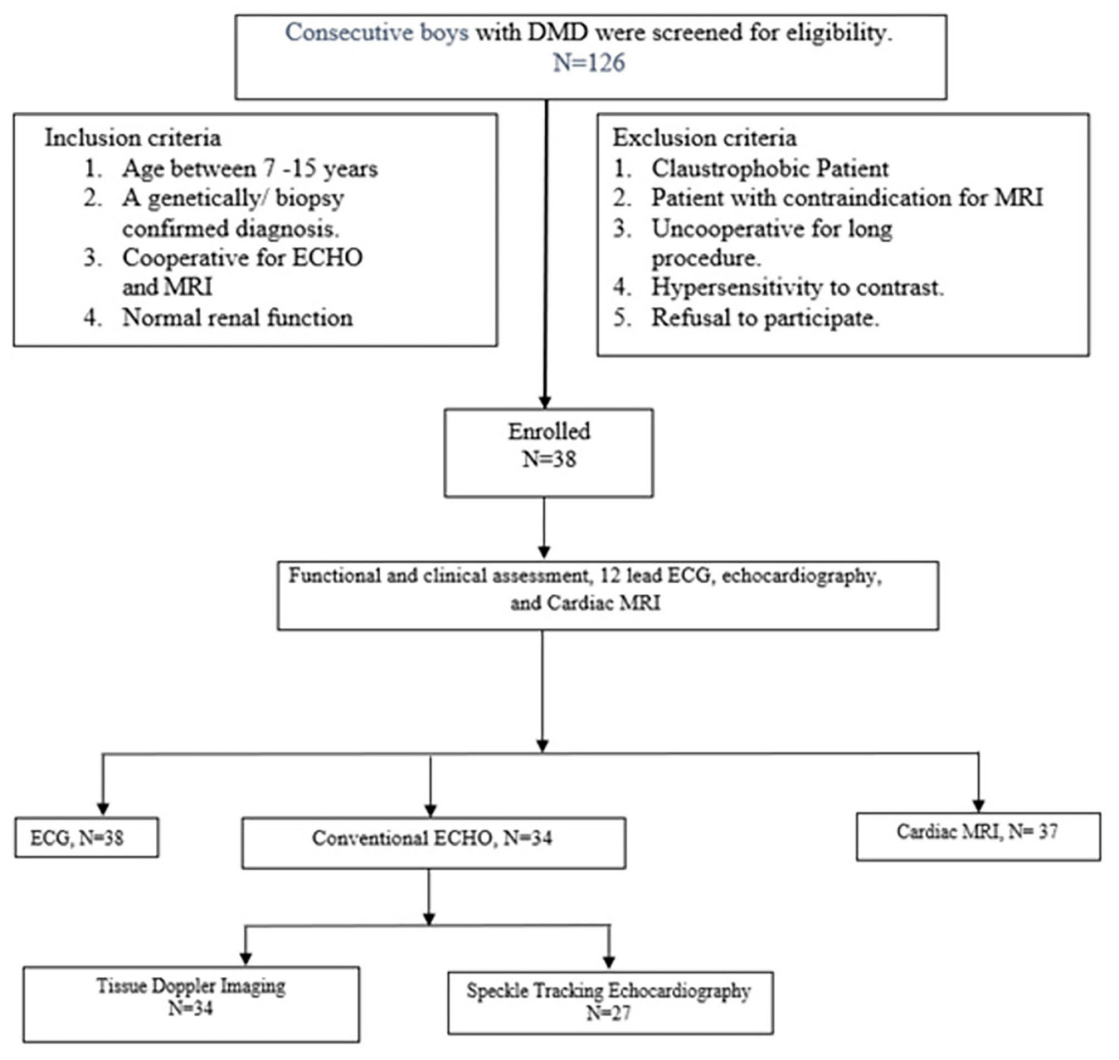
Study flow chart.

A total of 350 boys with DMD are registered in the pediatric neurology clinic; 126 boys visited the outpatient department during the study period and were screened for eligibility. Thirty-eight boys with DMD, who fulfilled the inclusion criteria were enrolled (study flow chart, Figure 1). The mean age was 8.8 ± 1.6 years, and median follow-up duration in the pediatric neurology clinic was 11.5 (3–22) months (Table 1). The diagnosis of DMD was confirmed with genetic testing in 36 boys and with muscle biopsy in two cases. At enrollment in the study, all boys with DMD were ambulatory and were independent in dressing, feeding, and toilet needs, and none was on respiratory support (Table 1). The mean 6 MWD was 371.6 ± 51 (mean, SD) m, 10-meter walk time was 11.1 (median, IQR 8.6–15) s. The upper limb Brooke score was 1 in all subjects, and the lower limb Vigno's score was 2 in 28 (74%); one each had Vigno's lower limb score of 3 and 4. Regarding clinical symptoms related to DMD-related cardiomyopathy, none was clinically symptomatic, or had peripheral edema or anasarca. The median resting heart rate was 94 (IQR 88–102) beats per minute, and 5(13%) cases had resting tachycardia.

**Table 1:** Demographic and clinical details of boys with DMD.

**Demographic profile**	***N* = 38**
Age, mean ± SD, years	8.78 ± 1.63
**Duration of follow-up in clinic, in months**	
Mean ± SD	16.47 ± 19.23
**Examination findings**	
**Anthropometry**	
Weight (kg), mean, SD	23 ± 6.3
Weight <2 z score, *n*, %	5 (13.21)
Height (cm), mean, SD	121 ± 8.3
Height < −2 z score, *n*, %	13 (84.21)
BMI (kg/m^2^), mean, SD	15.3 ± 3.6
BMI < −2 z score, *n*, %	6 (15.8)
**Cardiac examination**	
Baseline tachycardia (HR > 110), *n*, %	5 (13)
SBP in supine position (mm Hg)	100.63 ± 11.57
DBP in supine position (mm Hg)	60.39 ± 9.65
Postural hypotension, *n*, %	4 (10.5)
**Functional assessment**	
Gower's time (in s), median, IQR	11.3 (9.9–17)^*^
10 MWT (in s), median, IQR	11.1 (8.6–15)^*^
**Vigno's scale for lower extremity**, ***n*****, %**	
1	8 (21%)
2	28 (74%)
3	1 (2.6%)
4	1 (2.6%)
6-min walk distance in meters (6 MWT), mean ± SD	371.6 ± 51
4 stairs ascent time (5), median, IQR	5 (3.8–7.2)^*^
4 stairs descent time (5), median, IQR	3.9 (2.8–5.4)^*^
Ambulation	38 (100%)
**Diagnostic investigations**	
CPK total, median, interquartile range	9,823 (6,623–14,514)
**Diagnostic confirmation**, ***n*****, %**	
Genetic	36 (95%)
Muscle biopsy	2 (5%)
**Genetic testing**, ***n*****, %**	
Exon deletion (by MLPA)	31 (81.6%)
Point mutations (by NGS)	5 (13.2%)
**Electrocardiogram findings**, ***n*****, %**	
R > 4 mm in V1	29 (76)
Deep and narrow Q in V5, V6, and aVL	18 (47)
R/S > 1 in V1	20 (53)
R/S > 1 in V2	23 (60)
Right ventricular ECG changes	13 (34)
Biventricular ECG changes	18 (47)

*10 MWT, 10-meter walk time; BMI, body mass index; CPK, creatine phosphokinase; NYHA, New York Heart association; DBP, diastolic blood pressure; IQR, interquartile range; MLPA, multiplex ligation-dependent probe amplification; NGS, next-generation sequencing; SBP, systolic blood pressure; SD, standard deviation; DMD, Duchenne muscular dystrophy; ECG, electrocardiography*.

* *Skewed data*.

### Electrocardiography

A 12-lead ECG was performed in all 38 boys with DMD: 29 (76%) had R > 4 mm in the V1 lead, 20 (53%) had R/S > 1 in the V1 lead, and 23 (60.5%) boys had R/S > 1 in the V2 lead suggestive of right ventricular pathology. Eighteen (47%) children had deep and narrow Q wave in leads V5 and V6, and aVL suggestive of left ventricular involvement; biventricular pathology was present in 18 (47%) patients (Table 1).

### Echocardiography

Echocardiography (TTE and TDI) was performed in 34 (89%) subjects, and STE was performed in 27 (71%) subjects. The details of echocardiographic finding are presented in Table 2. The mean percentage EF in boys with DMD was 60 ± 4.13% (mean, SD) in TTE. The EF was ≤ 55% in 5 (15%) boys. Mean percentage FS was 31.5 ± 2.8 (mean, SD), and 3 (9%) subjects had an FS of ≤28%. LV wall thinning behind posterior mitral leaflet was seen in one subject. None had mitral regurgitation or aortic regurgitation.

**Table 2:** The details of echocardiography findings in boys with DMD.

**ECHO variables**	**Values**
**2D conventional echocardiography**	***N*** **=** **34**
**Ejection fraction (%)**	
Mean ± SD	60.04 ± 4.13
Normal (>55%), *n*, %	29 (85)
^#^Mildly abnormal (41–55), *n*, %	5 (15)
**LVID d (cm**)
Mean ± SD	36.85 ± 3.3
LVID d > 2 SD	0
**Percentage fractional shortening**	
Mean ± SD	31.52 ± 2.8
^$^ <28% fractional shortening, *n*, %	3 (9)
**MV-E-wave (cm/s)**	
Mean ± SD	107 ± 13.7
Presence of MR	0
Presence of AR	0
Presence of LV thinning behind posterior mitral leaflet, *n*, %	1 (2.9)
Presence of LV akinesia and/ or dyskinesia	1 (2.9%)
**Tissue doppler imaging parameters**	***N*** **=** **34**
**Early diastolic mitral annulus velocity Ea (m/s)**	
Median (IQR) 0.10 (0.07–0.14)	0.13 (0.12–0.14)
**Medial mitral annulus ratio Ea/Aa**	
Median (IQR) 6.67 (4.62–11.25)	8.3 (7.0–8.8)
**Speckle tracking echocardiography parameters**	***N*** **=** **27**
**End diastolic volume (ml/m** ^**2**^ **)**	
Mean ± SD (54 ± 10)	46.16 ± 18
**End systolic volume (ml/m**^**2**^)	
Mean ± SD (21 ± 5)	19.1 ± 6.6
**Ejection fraction (%)**	
Mean ± SD	58 ± 4.28
^**$**^**Global strain**, ***n*** **(%)**	
< -16 (abnormal)	1 (3.8)
−16 to −18 (borderline)	3 (11.5)
>-18 (normal)	22 (84)

*Fractional shortening cutoff 28% taken from meta-analysis done by Guang Song et al*.

# *Ejection fraction classification done on the basis of an Update from the American Society of Echocardiography and the European Association of Cardiovascular Imaging*.

$ *Global strain cutoff reference taken from the American College of Cardiology Foundation*.

^*^Skewed data.

*AR, aortic regurgitation; IQR, interquartile range; LV, left ventricle; LVIDd, left ventricle diameter in diastole; MR, mitral regurgitation; MV-E-wave, mitral valve flow E wave velocity; SD, standard deviation. Bold values represents the number of subjects with investigation*.

### Tissue Doppler Imaging

The median value of early diastolic flow velocity around the mitral annulus was 0.13 (IQR 0.12–0.14) cm/s, and the E/A ratio calculated for septal mitral annulus was 8.3 (7.0–8.8), and both the values were within the normative range (Table 2).

### Speckled Tracking Echocardiography

Global circumferential strain pattern was measured in 27 subjects, and a value of 16% was considered as abnormal, 16–18% as borderline, and >18% as normal. Twenty-two subjects (56.4%) had no evidence of global strain, three (7.7%) had borderline reduction in global strain, and global strain was significantly reduced in 1 (2.6%) subject (Table 2).

### Cardiac MRI

**Figure 2:**
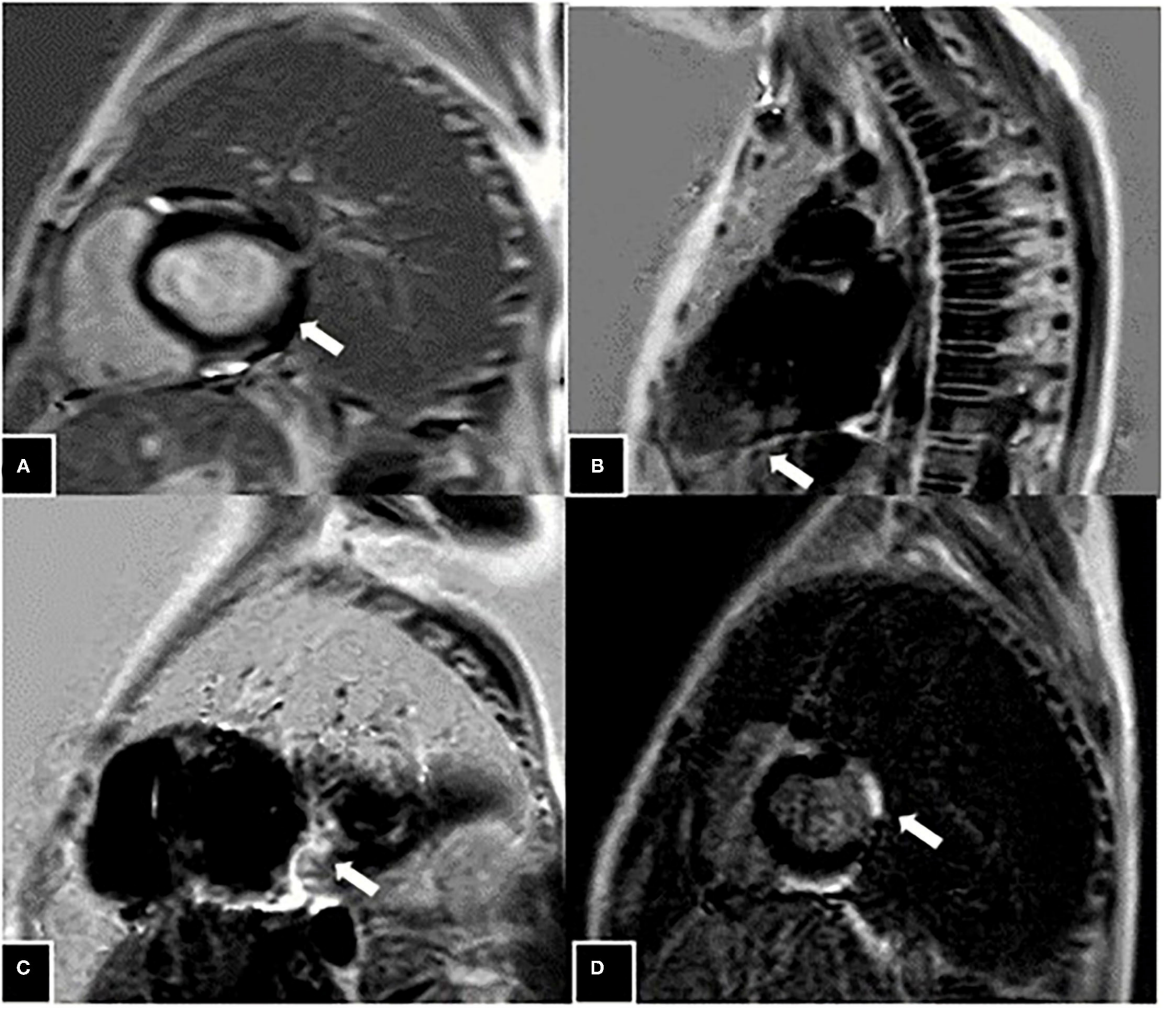
Grade 2 late gadolinium enhancement (LGE) (marked with white arrow) seen in the anterolateral wall at mid cavity level at the age of 8 years **(A)**, patchy areas of grade-1 LGE seen in the inferoseptal wall at mid cavity level at the age of 11 years **(B)**, patchy involvement (grade-0) seen in the form of LGE presence in the inferoseptal wall at mid cavity at age of 9 years **(C)**, and Grade 2 LGE seen in the anterolateral wall at the level of the base in an 8-year-old boy **(D)**.

Cardiac MRI were performed in 37 (97%) boys with DMD. The CMRI-derived EF of >55% (group I) was considered as normal myocardial contractility, and EF ≤ 55% (group II) was considered abnormal (Table 3). Twenty (53%) boys had ≤ 55% EF in the CMRI, and the mean EF in group II was 43.6 ± 5.6%. The Global left ventricular wall motion was reduced in 9 (23%) subjects, 7 (18%) had mild hypokinesia, and 2 (5%) had moderate hypokinesia. Segmental hypokinesia was observed in the basal and mid anteroseptal (26%), inferoseptal (10.5%), and mid inferolateral segments (8%). In CMRI, 4 (10.5%) subjects had LGE, 2 (5.3%) had grade-2 involvement of anterolateral wall at midcavity and basal level, grade-1 LGE in one (2.6%) at inferoseptal wall, and grade-0 involvement at mid inferoseptal segment in one subject (Figure 2).

**Table 3:** Details of cardiac MRI in boys with DMD.

**Variables**	***N* = 37**
**Resting cardiac function**, ***n*****, %**	
Group I EF ≥ 55%	17 (45)
Mean EF (mean ± SD)	64 ± 6.14
**Group II EF** **<** **55%**	20 (53)
Mean EF (mean ± SD)	43.60 ± 5.60
**Global left ventricular wall motion**	
**Likert scale**, ***n*****, %**	
1 (Normokinesia)	28 (73.7)
2 (Mild hypokinesia)	7 (18.4)
3 (Moderate hypokinesia)	2 (5.3)
**Most frequently involved cardiac segments in cardiac MRI**, ***n*****, %**	
Basal inferoseptal	5 (13.2)
Mid inferoseptal	4 (10.5)
Mid inferior	5 (13.2)
Mid inferolateral	4 (10.5)
Apical inferior	3 (7.9)
Apical lateral	3 (7.9)
**Tissue characteristic in cardiac MRI**, ***n*****%**	
Presence of late gadolinium enhancement (LGE)	4 (10.5)
Grade-0	1 (2.6)
Grade-1	1 (2.6)
Grade-2	2 (5.3)
**Segments showing LGE**	
Mid cavity	1 (2.6)
Mid inferoseptal	2 (5.3)
Mid anterolateral	1 (2.6)

The mean age, 6 MWD, TFTs, and Vigno's lower limb score were similar in both the CMRI-derived EF groups (Table 4). In group 2, with CMRI-derived EF <55%, a significantly higher number of subjects had left ventricular wall hypokinesia, and 9 (24%) had left ventricular wall motion abnormalities, in comparison with group 1, with EF > 55%, where none had left ventricular wall motion anomaly (*p*-value = 0.001). One subject in group 2, with CMRI-derived EF <55%, TTE also revealed left posterior wall hypokinesia. The E/A ratio, which was measured through TDI, was higher in group II (7.43 ± 1.13 vs. 8.45 ± 1.05, *p*-value of 0.01) (Table 4). Global strain pattern measured with STE was reduced among three subjects in group 2, with CMRI-derived EF ≤ 55% (*p*-value = 0.038). The correlation between the CMRI-derived EF and TTE-derived EF was weak (*r* = 0.234, *p*-value = 0.189). Similarly, the correlation between TTE-derived FS and CMRI-derived EF was non-significant. We did not find any significant correlation between the CMRI-derived ejection fraction and global strain pattern (*r* = 0.217, *p* > 0.05). According to the proposed DMD-related cardiomyopathy classification, in our cohort of 38 boys with DMD, 80% boys had stage 1, 5 (14.5%) had stage 2, and 2 (6%) had stage 3 DMD-related cardiomyopathy (Table 5).

**Table 4:** Comparison of the various functional parameters between the two groups divided according to the ejection fraction obtained from CMRI; group I with EF of ≥55% and group II with EF < 55%.

**Parameters**	**Group I** **(*n* = 17)**	**Group II** **(*n* = 20)**	***p*-value**
Age (years)	9.06 ± 1.1	9.25 ± 1.8	0.53
10 MWT (mean ± SD)	13.2 ± 6.9	11.3 ± 3.5	0.07
6 MWD (mean ± SD)	383 ± 55.6	360 ± 46.5	0.08^*^
**CMRI parameters**			
LV ejection fraction%	64 ± 6.14	43.60 ± 5.60	<0.001^*^
LV wall motion abnormality, *n*, %	0 (0%)	9 (23.7%)	0.001
Late gadolinium enhancement presence, *n*, %	1 (2.6%)	2 (5.3%)	0.37
**2D echocardiography parameters**			
LV ejection fraction ≥ 55%, *n*, %	36 (94.7)	2 (5.3)	0.98
LV posterior wall hypokinesia, *n*, %	0 (0%)	1 (2.6%)	0.06
**Tissue Doppler imaging diastolic function parameters**			
Medial mitral annulus Ea (m/s) (mean ± SD)	14.04 ± 1.74	12.55 ± 3.08	0.57^*^
E/Ea (mean ± SD)	7.43 ± 1.13	8.45 ± 1.05	0.014^*^
**Speckled tracking echocardiography**			
Global strain (*n* = 26) < -18%	0 (0%)	3 (7.9%)	0.038

* *Test applied: independent T-test*.

*10 MWT, 10-meter walk time; 6 MWD, 6 min walk distance; LV, left ventricle; E/Ea, early inflow velocity (E wave) to early to late (A wave) inflow Doppler velocity ratio; SD, standard deviation; 2D, two dimension; EF, ejection fraction; CMRI, cardiac MRI*.

**Table 5:** Duchenne muscular dystrophy-associated cardiomyopathy staging and number of boys with abnormal ECG, ECHO, and CMRI parameters.

**DMD associated cardiomyopathy staging**	**Number (percentage)**
Stage 1	28 (80%)
Stage 2	5 (14.5%)
Stage 3	2 (5.7%)
**Abnormal ECG** (*n* = 38)	31 (81.6%)
**Abnormal 2D-ECHO**	
LV posterior wall thinning/hypokinesia (*n* = 34)	2 (6%)
Fractional shortening <28% (*n* = 34)	3 (9%)
LV diastolic dysfunction in TDI (*n* = 34)	0
Abnormal global LV strain in STE (*n* = 26)	4 (12%)
**Abnormal CMRI (*****n*** **=** **37)**	
Ejection fraction <55%	20 (52.6%)
Mild to moderate hypokinesia	9 (23.7%)
Late gadolinium enhancement	3 (7.9%)

*DMD, Duchenne muscular dystrophy; ECG, electrocardiogram; ECHO, echocardiography; CMRI, cardiac MRI; LV, left ventricular; TDI, tissue Doppler imaging; STE, speckled tracking imaging*.

## Discussion

In this cross-sectional study, we evaluated the clinical features, echocardiography, and CMRI for the diagnosis of cardiomyopathy in early ambulatory boys with DMD. Clinically, none of the subjects had symptoms related to cardiac dysfunction. All boys were ambulatory, and none was receiving nocturnal or daytime CPAP/BIPAP respiratory support.

Our study supports the fact that CMRI has a better diagnostic yield for identification of preclinical or early features of DMD-related cardiomyopathy and is more sensitive for identification of subtle myocardial dysfunction, wall motion, and morphological abnormalities in comparison with conventional TTE. According to the CMRI, 53% boys with DMD had reduced EF, 24% had left ventricular wall motion abnormalities, and 10% had LGE, while only one subject (3%) had wall motion abnormality and 15% had ≤ 55% EF in TTE.

Presence of left ventricular wall motion abnormalities in CMRI has good predictive value for the detection of occult regional cardiac dysfunction (23). In our study, CMRI showed the presence of LGE in young boys with DMD, as early as 8 years of age, and was predominantly seen in inferoseptal segments and anterolateral segments. CMRI has the advantage of providing an accurate three-dimensional view of global and regional functions (24–28). The fibrotic area in the myocardium shows the presence of LGE due to decreased contrast washout. LGE is a sensitive marker of myocardial fibrosis and typically involves the basal inferolateral free wall. Authors have reported the presence of LGE in the subepicardium of the inferolateral wall secondary to increased mechanical stress at this region (21, 26, 29). In DMD-related cardiomyopathy, a distinct pattern of cardiac involvement is present, starting from subepicardial fibrosis of inferolateral segments and then progressing globally, although with scattered myocardial fibrosis and fatty infiltration that may be present anywhere not limited to posterolateral segments (5, 26, 30–33). Kan et al. demonstrated that age had a strong correlation with the presence of LGE, and boys with LGE positivity were significantly older and had decreased LVEF. As more younger boys were part of this study, LGE was seen in as young as 8.8 years of age (21, 24).

STE showed reduced global strain in one subject and borderline strain pattern in three subjects. Global strain is reported to have good diagnostic yield for the detection of myocardial fibrosis in children even <7 years of age (5, 26). Taqatqa et al. reported a reduced global circumferential strain, and Cho et al. reported a reduced global longitudinal strain in boys with DMD (34, 35). Subsequently a meta-analysis by Song et al. concluded that global strain pattern is the best technique for early detection of myocardial strain (36). Global strain was reduced in 4 (15%) subjects in our cohort. It suggests that the microfibrosis was present throughout the myocardium suggesting global strain pattern as a valuable marker for the detection of subclinical involvement in boys with DMD (5, 26). Previously, it was thought that the myocardium is rarely affected by fibrosis prior to the age of 10 years; in our study, reduced global strain in four subjects suggests the presence of microfibrosis in the myocardium (26, 37). Cho et al. and Markham et al. demonstrated that diastolic dysfunction precedes the development of overt systolic dysfunction in DMD-related cardiomyopathy (35, 38, 39). Contrary to the above studies, diastolic abnormalities were not detected in TDI in any subject in our cohort.

A poor correlation between the he CMRI-derived EF and echo-derived EF and other parameters was observed in this study. Previously, Soslow et al. (20) correlated the echo and CMRI in boys with DMD, and only a modest correlation between CMRI-derived EF and fractional shortening in echocardiography was observed. Due to poor window, TTE is not able to visualize 69 out of 204 cardiac segments, though with three-dimensional view, CMRI can visualize all segments. Mid inferolateral and inferoseptal segments showed wall hypokinesia in the CMRI (20). CMRI offers the advantage of an excellent anatomical view of all the cardiac chambers with morphological and functional characteristics of the myocardium; the limitation is that the cost of CMRI is high, and it requires a prolonged imaging acquisition time and marked patient cooperation. The other practical limitation in the routine use of CMRI among boys with DMD is that facility and expertise for CMRI may not be available at all centers. Though TTE is a standard technique for cardiac screening of DMD-related cardiomyopathy, it lacks the abilities to detect early myocardial fibrosis and visualize all cardiac segments. The size of the left ventricle and contractility remains normal until widespread myocardial fibrosis is established.

In the absence of clinical features related to cardiac dysfunction, routine cardiovascular screening becomes especially important for the early detection of DMD-related cardiomyopathy. The DMD care guidelines 2018 suggested the use of CMRI for early detection of DMD-related cardiomyopathy and initiation of angiotensin-converting enzyme (ACE) inhibitors in boys > 10 years of age or those who have cardiac dysfunction in the investigations (8). We initiated ACE inhibitors to all boys who had evidence of myocardial dysfunction in CMRI and TTE. Long-term clinical and CMRI follow-up is required to capture the progression in cardiomyopathy in these boys.

An adequate sample size, detailed cardiac evaluation, and comparison of CMRI and echocardiographic findings in young boys with DMD are some of the strengths of this study. The use of standard CMRI and echocardiographic techniques increases the study's internal and external validity. Inability to perform STE in all subjects and all three echocardiographic parameters among all who underwent CMRI was a limitation of this study. Lack of evaluation for GLS with STE among one-third of the subjects could have introduced bias in the study.

To conclude, cardiomyopathy remains clinically silent in early ambulatory boys with DMD. The CMRI has a higher sensitivity for early diagnosis of DMD-related cardiomyopathy with reduced contractility, wall hypokinesia, and LGE, in comparison with conventional echocardiography.

## Data Availability Statement

The raw data supporting the conclusions of this article will be made available by the authors, without undue reservation.

## Ethics Statement

The studies involving human participants were reviewed and approved by Institute Ethics Committee, PGIMER, Chandigarh. Written informed consent to participate in this study was provided by the participants' legal guardian/next of kin.

## Author Contributions

NP: patient enrollment and data analysis. RS: study planning, design, data analysis, wrote the manuscript, and final approval for publication. BKS: performed echocardiography and echo data interpretation. UD: CMRI data analysis. RMK: guidance for echocardiographic evaluation and data interpretation. NS: study design, critical review of the manuscript, and final approval for publication. All authors contributed to the article and approved the submitted version.

## Conflict of Interest

The authors declare that the research was conducted in the absence of any commercial or financial relationships that could be construed as a potential conflict of interest.

## Publisher's Note

All claims expressed in this article are solely those of the authors and do not necessarily represent those of their affiliated organizations, or those of the publisher, the editors and the reviewers. Any product that may be evaluated in this article, or claim that may be made by its manufacturer, is not guaranteed or endorsed by the publisher.
